# Efficacy and Safety of Transphyseal Screw Hemiepiphysiodesis for Lower Limb Deformity Correction in Pathological Bone Conditions: A Systematic Review

**DOI:** 10.7759/cureus.115434

**Published:** 2026-08-30

**Authors:** Rami Rajjoub, Mutaleeb A Shobode, Aaron DeVilbiss, Mubashir Wani, Kirsten Tulchin-Francis, Sean A Tabaie

**Affiliations:** 1 Orthopedic Surgery, Nationwide Children's Hospital, Columbus, USA; 2 Orthopedics, Nationwide Children's Hospital, Columbus, USA

**Keywords:** distal femoral physis, growth modulation, limb-length discrepancy, pediatric orthopedics, skeletally immature, transphyseal screws

## Abstract

Transphyseal screw hemiepiphysiodesis is a minimally invasive technique for guided growth correction of angular deformities and limb-length discrepancies of the lower extremities in the pediatric population. Pathological bone conditions, including Blount disease, hereditary multiple exostoses (HME), cerebral palsy (CP), and other neuromuscular disorders, present unique challenges that may affect correction rates, rebound risk, and postoperative complications. The purpose of this study is to review the outcomes and complications of transphyseal screw hemiepiphysiodesis in skeletally immature patients with defined pathological bone conditions. In accordance with Preferred Reporting Items for Systematic Reviews and Meta-Analyses (PRISMA) guidelines (PROSPERO ID: CRD420261426720), a systematic search of PubMed, Embase, and the Cochrane Library was conducted from inception to May 2026. Inclusion required a defined pathological etiology, transphyseal screw intervention, at least five patients or limbs, a quantitative radiographic outcome, and a minimum of six months of follow-up, with study quality assessed using the methodological index for non-randomized studies (MINORS) criteria. Twenty studies encompassing 657 patients and 1,072 limbs/segments were ultimately included and grouped into four anatomic categories: ankle/distal tibia (9 studies, 358 patients), knee coronal plane (4 studies, 121 patients), knee sagittal plane (4 studies, 113 patients), and hip (3 studies, 65 patients). Ankle valgus correction rates ranged from 0.24° to 0.90°/month across pathological diagnoses, with significantly faster rates in CP than in spina bifida or HME. For coronal knee deformity in Blount disease, the mean mechanical axis deviation correction rate was 3.66 ± 1.51 mm/month, and 90% surgical success was achieved in adolescent tibia vara. Additionally, coxa valga in CP improved, with mean head-shaft angle reductions of 13°-14° over two years. Complications were predominantly hardware-related, including difficult screw extraction, the physis growing off the screw, and rebound valgus in several ankle cases depending on etiology. In conclusion, transphyseal screw hemiepiphysiodesis is effective across a broad spectrum of pathological bone conditions, producing statistically and clinically significant deformity correction at the ankle, knee, and hip. However, correction rates vary meaningfully by etiology, hardware issues remain the primary complication, and rebound deformity remains a distinct concern after premature screw removal, particularly in patients with HME and CP.

## Introduction and background

Temporary epiphysiodesis has become a widely used strategy for the correction of angular limb deformities and limb-length discrepancies in skeletally immature patients [1,2]. This technique relies on the Hueter-Volkmann principle, where asymmetric compression applied across the growth plate slows growth on the tethered side while allowing the contralateral physis to grow unrestricted [3]. Early interventions for limb-length discrepancies used permanent epiphysiodesis via the open Phemister or percutaneous Bowen techniques; however, these irreversible procedures require precise timing to avoid over- or under-correction [4,5]. Physeal stapling offered a reversible alternative but was associated with its own set of complications, including staple breakage, migration, and physeal arrest [6].

Métaizeau et al. introduced percutaneous epiphysiodesis using transphyseal screws (PETS), placing single-threaded cannulated screws obliquely across the physis [7]. The advantages of this technique included minimal soft tissue dissection, immediate postoperative weight-bearing, and the reversibility of growth inhibition upon screw removal [7]. Since then, the technique has been adapted across the lower extremities to treat coxa valga at the proximal femur in cerebral palsy (CP), sagittal-plane flexion contractures at the anterior distal femur, and coronal-plane knee deformities at the lateral proximal tibia and distal femur [8-10].

Despite the increased utilization of PETS, the current literature lacks outcome data in children with Blount disease, hereditary multiple exostoses (HME), osteogenesis imperfecta (OI), and neuromuscular disorders. For instance, two recent systematic reviews and meta-analyses evaluated PETS for limb-length discrepancy and coronal knee deformities but predominantly included idiopathic patient populations [11,12]. The aforementioned pathological bone conditions present with fundamentally different biological environments for guided growth and may result in different outcomes when compared to idiopathic cohorts [13-15].

The purpose of this systematic review was to synthesize the available clinical evidence for transphyseal screw hemiepiphysiodesis specifically in pediatric patients with pathological bone conditions, across all anatomic sites. Specifically, we sought to: (1) quantify the rates of angular correction and overall surgical success across distinct pathological diagnoses; (2) evaluate the incidence and nature of postoperative complications; (3) determine the risk of rebound deformity following hardware removal; and (4) identify clinical or radiographic factors that modify these outcomes.

## Review

Methods

Search Strategy

A comprehensive electronic search of PubMed/MEDLINE, Embase (via Ovid), and the Cochrane Central Register of Controlled Trials (CENTRAL) was performed from database inception to May 31, 2026. The search strategy combined three conceptual domains using Boolean logic: (1) implant-specific terminology (e.g., transphyseal screw and PETS), (2) procedural terminology (e.g., hemiepiphysiodesis, guided growth, and growth modulation), and (3) pathological etiologies (e.g., Blount disease, skeletal dysplasia, CP, and OI). Concepts were combined using the framework (implant terms OR procedure terms) AND pathological etiologies without language restrictions.

Eligibility Criteria

Studies were eligible if they met all of the following inclusion criteria: (1) study design included randomized controlled trials, prospective or retrospective cohort studies, or case series with greater than or equal to five patients or limbs treated with transphyseal screws; (2) patient population consisted of skeletally immature patients with a clearly defined pathological bone condition; (3) intervention was transphyseal screw hemiepiphysiodesis or epiphysiodesis at any anatomic site; (4) at least one quantitative radiographic outcome measure was reported; (5) follow-up was minimum six months or to hardware removal/skeletal maturity; and (6) full-text was in English.

Studies were excluded if they: (1) reported exclusively idiopathic deformities without a separable pathological subgroup; (2) used only tension-band plates, staples, or permanent Phemister-type epiphysiodesis without a transphyseal screw group; (3) included fewer than five patients or limbs; (4) were animal, cadaveric, or biomechanical studies or review articles; (5) provided no quantitative radiographic outcome data.

Study Selection

All search results were imported into reference management software, and duplicates were removed. The first and second reviewers independently screened titles and abstracts against the eligibility criteria. Full texts of potentially eligible studies were retrieved and independently assessed by both reviewers. Disagreements were resolved by consensus. Where only a comparative study's transphyseal screw arm met inclusion criteria (e.g., screw vs. tension-band plate comparisons), data were extracted from the screw arm only.

Data Extraction

Two reviewers independently extracted data using a standardized extraction form, including: study characteristics (author, year, country, design, level of evidence, sample size, and follow-up duration); patient demographics (age, sex, BMI, underlying diagnosis, and Gross Motor Function Classification System (GMFCS) level for patients with CP); intervention details (screw type, diameter, trajectory/direction, anatomic location, number of screws per segment, and concurrent procedures); and outcome data (preoperative and postoperative angular measurements, correction rate in degrees or millimeters per month, time to correction, complications, rebound deformity, and secondary procedures). Functional outcome data were extracted where reported. Original author-defined outcome definitions (e.g., criteria for success, failure, complication severity, and rebound deformity) were retained as reported in the primary studies to prevent misclassification bias. Data were analyzed on an available-case basis without statistical imputation; when specific variables or numerical outcomes were absent, they were documented as "not reported" across summary tables, and studies were retained for all other reported metrics.

Quality Assessment

Two reviewers independently assessed methodological quality using the methodological index for non-randomized studies (MINORS) criteria [16]. Each item was scored 0 (not reported), 1 (reported but inadequate), or 2 (reported and adequate). Discrepancies were resolved by consensus.

Results

Study Selection

The literature search identified a total of 528 studies, yielding 520 for title and abstract screening after removal of duplicates. After initial title and abstract screening, 36 full-text articles were selected and reviewed. After applying inclusion and exclusion criteria, 20 studies were included in the final review.

**Figure 1:**
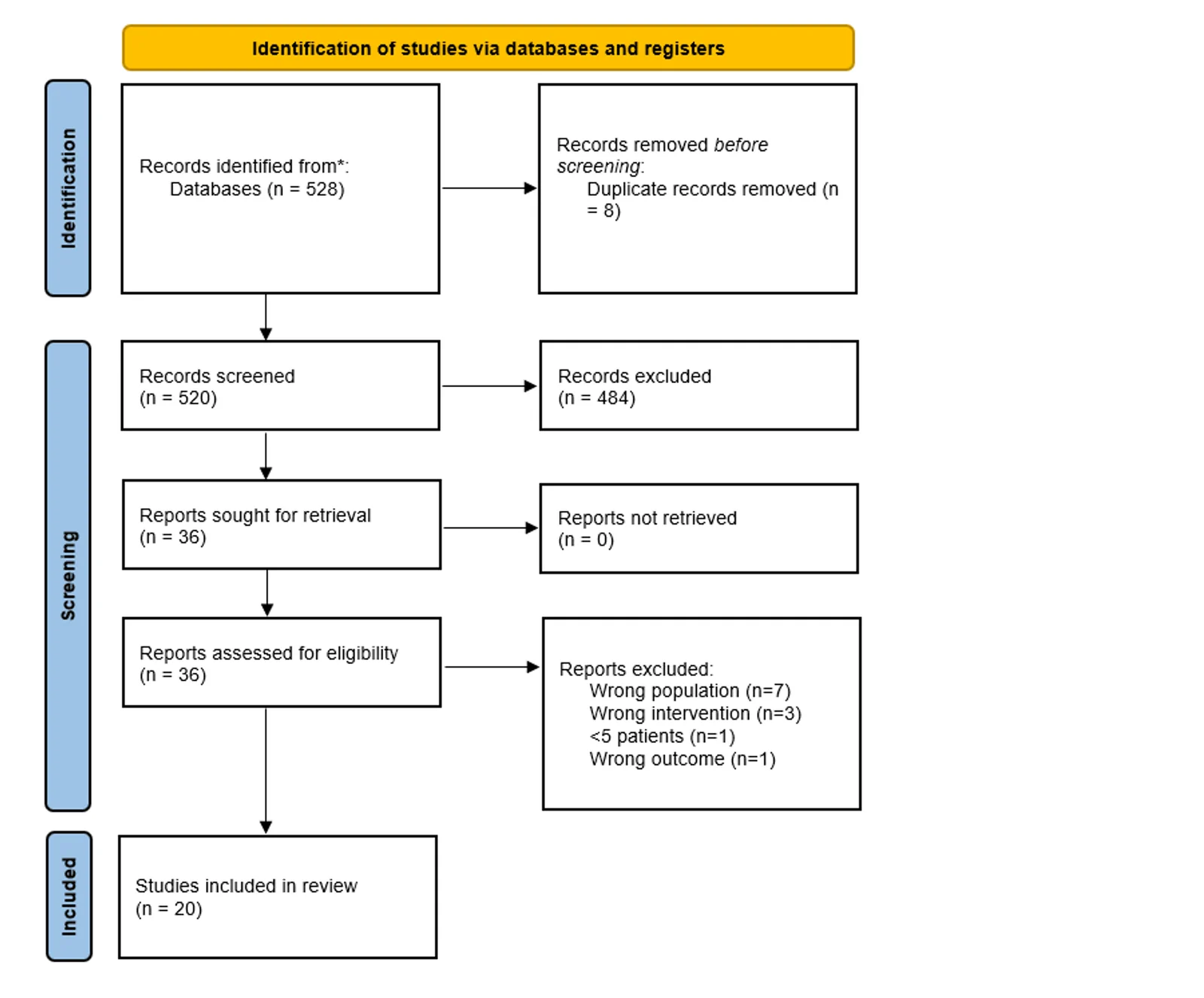
PRISMA Flow Diagram * Represents database searching of PubMed/MEDLINE, Embase, and the Cochrane CENTRAL. Preferred Reporting Items for Systematic Reviews and Meta-Analyses (PRISMA) flow diagram illustrating the study selection process. A total of 528 records were identified through database searching of PubMed/MEDLINE, Embase, and the Cochrane CENTRAL.

The most common reasons for exclusion were study reporting exclusively idiopathic deformity without a pathological subgroup (n=7), use of tension-band plates or staples only without a transphyseal screw group (n=3), fewer than five patients (n=1), and the primary outcome being intra-articular deformity morphology rather than angular correction (n=1) (Figure 1).

Study Characteristics

The 20 included studies were published between 1997 and 2026 and originated from 8 countries [8,9,14,15,17-32]. They encompassed a total of 657 patients and 1,072 limbs or segments. Study designs included 1 retrospective comparative cohort study (level II), 1 prospective case series (level IV), 6 retrospective cohort studies (level III), and 12 retrospective case series (level IV). No randomized controlled trials were identified.

Mean follow-up ranged from 10.5 to 65 months after hardware removal. The age at surgery ranged from a mean of 6.2 years to 13.8 years. MINORS scores for non-comparative studies ranged from 8 to 12 of a possible 16; comparative studies scored 16 to 19 of a possible 24. Detailed study characteristics are presented in Tables 1, 2.

**Table 1:** Characteristics of the Included Studies Summary of the 20 included studies detailing first author, year of publication, country of origin, study design, level of evidence, number of patients and limbs or segments, underlying pathological diagnoses, anatomic site of intervention, and mean follow-up duration. CP, cerebral palsy; FSP, familial spastic paraparesis; GMFCS, Gross Motor Function Classification System; HME, hereditary multiple exostoses; IQR, interquartile range; LoE, level of evidence; MED, multiple epiphyseal dysplasia; MMC, myelomeningocele; NF1, neurofibromatosis type 1; OI, osteogenesis imperfecta; Pts, patients; SD, standard deviation.

Study	Country	Design	LoE	# Pts	# Limbs	Anatomic site	Etiology	Mean age ± SD	Follow-up
Ankle									
Aurégan et al., 2011 [17]	France	Retrospective case series	IV	7	10	Distal tibia/medial malleolus	HME 4 (6); NF1 2 (2); Larsen 1 (2)	11 (range 9.5-15)	To skeletal maturity
Chang et al., 2015 [19]	USA	Retrospective	IV	37	63	Distal tibia/medial malleolus	HME 9 (14); CP 2 (4); spina bifida 5 (7); idiopathic 12 (24); other 9 (14)	11.0 ± 1.9	1.6 y (to removal)
Driscoll et al., 2014 [20]	USA	Retrospective comparative	II	24	35	Distal tibia/medial malleolus	HME 12; bone dysplasia 12; myelodysplasia 5; fibular deficiency 3; other 3	9.8 (range 4.5-14.5)	34 mo
Dussa et al., 2024 [21]	Germany	Retrospective	III	104	155	Distal tibia/medial malleolus	Idiopathic 46; CP 27; MMC 43; HME 18; bone dysplasia 21	10.9 ± 1.4	To end of growth
Franzone et al., 2022 [14]	USA/Croatia	Retrospective multicenter case series	IV	18	12	Distal tibia/medial malleolus	Osteogenesis imperfecta (I 5, III 4, IV 6, V 3)	11.9 ± 2.3	3.1 y
Rupprecht et al., 2015 [28]	Germany	Retrospective case series	IV	12	21	Distal tibia/medial malleolus	HME (all)	11.6 ± 1.5	22 mo after removal
Stevens and Belle, 1997 [30]	USA	Retrospective case series	IV	31	50	Distal tibia/medial malleolus	Neuromuscular 18; skeletal dysplasia 5; chromosomal 3; HME 2; limb hypoplasia 2; tibial pseudarthrosis	11.8 (range 6.6-15.4)	4.4 y
Westberry et al., 2020 [31]	USA	Retrospective case series	IV	119	189	Distal tibia/medial malleolus	Clubfoot 48 (71); other 33 (53); CP/FSP 18 (29); syndrome/dysplasia 10 (20); HME 10 (16)	11.7 (range 5.4-15.7)	4.2 y
Yilmaz et al., 2014 [32]	USA/Croatia	Retrospective case series	IV	6	12	Distal tibia/medial malleolus	Skeletal dysplasia	10.0 ± 2.9	25 mo
Knee Coronal Plane									
Braga et al., 2022 [18]	Brazil	Retrospective case series	IV	13	20	Proximal tibia (lateral)	Adolescent tibia vara (late-onset Blount)	12.8 (range 10.1-15.1)	65 mo
Khoury et al., 2007 [23]	USA	Retrospective	IV	30	66	Distal femur ± proximal tibia	Blount 6; fibular hemimelia 4; MED 2; neuromuscular 2; OI 1; fibrous dysplasia 1; osteochondroma 1; idiopathic 13	12.4-13.9	To maturity/removal
Murphy et al., 2020 [26]	USA	Retrospective	IV	9	14	Proximal tibia ± distal femur	Juvenile and adolescent tibia vara (Blount)	10.4 (range 8.3-12.8)	23 mo
Tageldeen et al., 2026 [15]	Egypt	Prospective case series	IV	61	80	Proximal tibia (lateral)	Blount (juvenile 64; adolescent 16 knees)	7.4 ± 2.2	10.5 mo (after removal)
Knee Sagittal Plane									
Krajewski et al., 2025 [24]	USA	Retrospective comparative	III	28	53	Distal femur (anterior)	CP 27; Aicardi-Goutières 1	9.8 ± 2.3	≥2 y
Long et al., 2020 [9]	USA	Retrospective case-control	III	10	20	Distal femur (anterior)	CP (all)	11.7 ± 2.6	26 mo
Nazareth et al., 2020 [27]	USA	Retrospective comparative	III	47	82	Distal femur (anterior)	CP 34; MMC 4; arthrogryposis 4; other 5	12.1 ± 2.7	1.0-4.6 y
Seth et al., 2024 [29]	Canada/USA	Retrospective case series	IV	36	68	Distal femur (anterior)	CP 25; MMC 3; syndrome 6; other 2	11.6 ± 2.1	24 mo
Hip									
Hsieh et al., 2019 [8]	Taiwan	Retrospective	III	24	48	Proximal femur	CP (all)	8 (range 5-12)	50 mo
Hsu et al., 2020 [22]	Taiwan	Retrospective comparative	III	32	61	Proximal femur	CP (all)	7 (IQR 6–9)	≥2 y
Lee et al., 2016 [25]	Taiwan	Retrospective case series	IV	9	13	Proximal femur	Spastic CP, GMFCS IV-V (all)	6.2 (range 4.1-9.3)	45.6 mo

**Table 2:** Patient Demographics and Intervention Details Patient demographics, including age at surgery, sex distribution, and body mass index, along with intervention details, including screw type, screw diameter, direction of insertion, number of screws per segment, and concurrent surgical procedures performed, were reported. alt., alternative; AO, Arbeitsgemeinschaft für Osteosynthesefragen (Association for the Study of Internal Fixation); GMFCS, Gross Motor Function Classification System; HSL, hamstring lengthening; NA, not applicable; NR, not reported; scr, screw; SS, stainless steel.

Study	Sex (M/F)	GMFCS	Screw type	Diameter (mm)	Entry/direction	Screws/segment	Concurrent procedures
Ankle Distal Tibia							
Aurégan et al., 2011 [17]	4/3	NA	Cortical, no flange	4.5	Medial malleolus, oblique coronal	1	None
Chang et al., 2015 [19]	16/21	NA	Partially threaded cannulated	4.0	Transphyseal medial malleolar	1	None
Driscoll et al., 2014 [20]	19/16	NA	Cannulated	4.5	Medial malleolar	1	Varied
Dussa et al., 2024 [21]	70/34	NA	Fully threaded SS cannulated	NR	Medial distal tibia	1	Foot deformity (2nd sitting)
Franzone et al., 2022 [14]	13/5	NA	Cannulated	4.5	Medial malleolar	1	Bisphosphonates (all)
Rupprecht et al., 2015 [28]	10/2	NA	Cortical (cannulated alt.)	4.5	Medial malleolus	1	None
Stevens and Belle, 1997 [30]	15/16	NA	Cannulated (AO)	4.5	Vertical, malleolar tip	1	8 pt (femoral stapling/subtalar fusion)
Westberry et al., 2020 [31]	84/35	NA	Fully threaded cannulated	4.0/3.5/4.5	Medial malleolar	1	None
Yilmaz et al., 2014 [32]	18/11	NA	Cannulated	NR	Medial malleolus	1	None (knee plates separate)
Knee Coronal Plane							
Braga et al., 2022 [18]	8/5	NA	Fully/long-threaded SS cannulated	7.0/6.5	Lateral proximal tibia, diagonal	1	None
Khoury et al., 2007 [23]	NR	NA	Fully threaded cannulated	7.3	Distal femur (uncrossed)/proximal tibia (crossed)	1-2	None
Murphy et al., 2020 [26]	6/3	NA	Fully threaded cannulated	6.5/7.3	Lateral proximal tibia, ante-/retrograde	1	4 limbs + distal femur
Tageldeen et al., 2026 [15]	26/35	NA	Fully threaded cannulated	6.5/4.0	Medial → lateral transphyseal	1	None
Knee Sagittal Plane							
Krajewski et al., 2025 [24]	17/11	NA	Fully threaded cannulated	4.0	Anterior distal femur, ante-/retrograde	2	Multiple (Botox, HSL, gastroc recession)
Long et al., 2020 [9]	4/6	II 4/III 6	Fully threaded cannulated	NR	Anterior distal femur	2	HSL + serial casting (all)
Nazareth et al., 2020 [27]	33/14	I 3/ II 6/ III 13/IV 12	Fully threaded cannulated	6.5 (1-scr)/4.5 (2-scr)	Anterior distal femur	1 or 2	HSL 24 (51%), others
Seth et al., 2024 [29]	20/16	II 10/ III 7/IV 9/V 3	Fully threaded cannulated	4.5	Anterior distal femur, antegrade	2	None
Hip							
Hsieh et al., 2019 [8]	17/7	I 3/II 4/III 7/IV 7/V 3	Fully threaded (Acutrak) or partially threaded (Synthes)	6.0/7.0	Inferomedial proximal femoral physis	1	Adductor tenotomy (24 hips)
Hsu et al., 2020 [22]	23/9	I 4/II 6/III 10/IV 9/V 3	Fully/partially threaded cannulated	6.0/7.0	Medial half of physis	1	Adductor tenotomy (all)
Lee et al., 2016 [25]	4/5	IV 6/V 3 (children)	SS cannulated	7.0	Eccentric, medial-third epiphysis	1	Soft tissue release (all)

Studies were distributed across four anatomic groups: (1) ankle/distal tibial valgus correction via the medial malleolar or transphyseal medial malleolar screw (TMMS) technique [14,17,19-21,28,30-32]; (2) coronal plane knee deformity (three Blount disease and one mixed) [15,18,23,26]; (3) sagittal plane knee flexion contracture via anterior distal femoral hemiepiphysiodesis (ADFH) [9,24,27,29]; and (4) proximal femoral coxa valga [8,22,25]. Pathological diagnoses represented within the study included Blount/tibia vara (5 studies), CP (14 studies), HME (4 studies), myelomeningocele (MMC)/spina bifida (6 studies), skeletal dysplasia (4 studies), OI (1 study), and mixed or other diagnoses (7 studies).

Ankle and Distal Tibial Valgus

Nine studies reported outcomes of TMMS or hemiepiphysiodesis for ankle valgus deformity, collectively including 358 patients and 547 ankles. The mean age at surgery ranged from 9.8 to 13.9 years (Table 3).

**Table 3:** Radiographic Outcomes Preoperative and postoperative radiographic measurements, correction magnitudes, correction rates, and author-defined surgical success rates organized by anatomic subgroup (ankle/distal tibia, knee coronal plane, knee sagittal plane, and hip). AE, anterior epiphysiodesis; CP, cerebral palsy; FTA, femorotibial angle; HME, hereditary multiple exostoses; HSA, head-shaft angle; LDF, lateral distal femoral; LDTA/mLDTA, (mechanical) lateral distal tibial angle; MAD, mechanical axis deviation; MMS, medial malleolar screw; MMC, myelomeningocele; MPTA, medial proximal tibial angle.

Study	Subgroup	Measure (unit)	Preop	Postop	Change (Δ)	Correction rate
Ankle						
Aurégan et al., 2011 [17]	Overall	Tibiotalar angle (°)	17.5	5	~12.5	—
Chang et al., 2015 [19]	Overall	Tibiotalar angle (°)	10.6 ± 4.3	3.1 ± 6.3	7.5 ± 7.6	0.37 ± 0.04°/mo
	HME	Tibiotalar angle (°)	—	—	—	0.32 ± 0.06°/mo
	Cerebral palsy	Tibiotalar angle (°)	—	—	—	0.90 ± 0.17°/mo
	Spina bifida	Tibiotalar angle (°)	—	—	—	0.24 ± 0.06°/mo
Driscoll et al., 2014 [20]	MMS arm	Tibiotalar angle (°)	77.1 ± 8.0	87.8 ± 11.6	10.7 ± 7.9	0.55 ± 0.41°/mo
Dussa et al., 2024 [21]	Overall	LDTA (°)	80.2 ± 2.1	87.0 ± 2.0	6.6 ± 1.0	0.45 ± 0.08°/mo
	CP	LDTA (°)	81.1 ± 3.9	87.7 ± 4.2	6.6 ± 4.8	0.54 ± 0.46°/mo
	MMC	LDTA (°)	78.3 ± 5.3	87.7 ± 6.1	9.4 ± 5.8	0.56 ± 0.37°/mo
	HME	LDTA (°)	79.8 ± 3.8	85.3 ± 5.6	5.5 ± 3.7	0.39 ± 0.33°/mo
	Bone dysplasia	LDTA (°)	78.3 ± 5.1	84.8 ± 5.4	6.5 ± 6.3	0.43 ± 0.37°/mo
Franzone et al., 2022 [14]	Screw subgroup	LDTA (°)	73.3 ± 3.4	86.0 ± 7.8	12.7 ± 8.5	0.7°/mo
Rupprecht et al., 2015 [28]	At screw removal	Tibiotalar tilt (°)	13.2 ± 4.9	0.8 ± 4.8	12.4 ± 6.9	0.63 ± 0.28°/mo
Stevens and Belle, 1997 [30]	Overall	Talar tilt (°)	—	—	9.7 (2-25)	—
Westberry et al., 2020 [31]	Overall	LDTA (°)	81.3	91.1	9.8	—
Yilmaz et al., 2014 [32]	Screw subgroup	mLDTA (°)	73.9 ± 8.7	86.1 ± 6.8	12.2 ± 4.5	—
Knee Coronal Plane						
Braga et al., 2022 [18]	Overall	MPTA (°)	79.0 ± 3.3	88.5 ± 3.7	9.5 ± 5.0	—
	Overall	FTA (°)	−11.0 ± 5.4	3.0 ± 4.2	14.0 ± 6.8	—
Khoury et al., 2007 [23]	Distal femur (pooled)	Correction (°)	—	—	6.91 ± 3.75	0.75 ± 0.45°/mo
	Proximal tibia (pooled)	Correction (°)	—	—	3.88 ± 3.57	0.37 ± 0.34°/mo
	Adolescent Blount	Correction, tibia (°)	—	—	—	0.79°/mo
Murphy et al., 2020 [26]	Overall	MAD (mm)	49	0	49	—
	Overall	MPTA (°)	81	92	11	—
Tageldeen et al., 2026 [15]	Overall	MAD (mm)	43.8 ± 15.1	0.7 ± 5.2	43.1 ± 14.9	3.66 ± 1.51 mm/mo
	Overall	MPTA (°)	77.0 ± 4.5	86.3 ± 2.4	9.3 ± 4.2	—
Knee Sagittal Plane						
Krajewski et al., 2025 [24]	Retrograde (clinical)	Passive knee extension (°)	−13.3 ± 6.9	−4.5 ± 6.1	8.6 ± 8.7	—
	Antegrade (clinical)	Passive knee extension (°)	−13.4 ± 8.5	−7.0 ± 5.7	6.4 ± 7.3	—
Long et al., 2020 [9]	AE group	Post. distal femoral angle (°)	88.2	109.1	20.9	~9.6°/y
	AE group (clinical)	Knee flexion contracture (°)	−21.7 ± 9.5	−3.8 ± 10.5	17.9 ± 16.6	—
Nazareth et al., 2020 [27]	2-screw	Knee flexion contracture (°)	−19 ± 7	−8 ± 8	11 ± 10.6	0.9 ± 0.8°/mo
	2-screw	Femoral physeal angle (°)	86.6 ± 8.5	75.7 ± 10.5	10.9 ± 13.5	—
Seth et al., 2024 [29]	Overall (24 mo)	LDF physeal angle (°)	93.0 ± 4.0	104.6 ± 6.3	11.6 ± 7.5	—
Hip						
Hsieh et al., 2019 [8]	Overall	HSA (°)	163 ± 6	150 ± 9	13 ± 7	—
	Overall	Migration percentage (%)	39 ± 10	29 ± 17	10 ± 11	—
Hsu et al., 2020 [22]	Group 1 (medial)	HSA (°)	163.6	149.7	13.9	—
	Group 1 (medial)	Migration percentage (%)	28.7	23.8	4.9	—
	Group 2 (middle)	HSA (°)	161.8	153.1	8.7	—
Lee et al., 2016 [25]	Overall (2 y)	HSA (°)	174.6	162.7	11.9	—
	Overall (2 y)	Migration percentage (%)	52.2	37.1	15.1	—

Stevens and Belle reported a mean talar tilt correction of 9.7 degrees (range: 2-25 degrees) across a heterogeneous pathological population (CP, myelodysplasia, HME, skeletal dysplasia, and chromosomal anomalies) with an average follow-up of 4.4 years [30]. Aurégan et al. confirmed valgus correction in 9 of 10 ankles across three pathological diagnoses (HME, neurofibromatosis type 1, and Larsen syndrome) with no complications [17]. Rupprecht et al. reported a mean correction rate of 0.63 ± 0.28 degrees/month in 12 patients with HME, achieving near-normalization of the tibiotalar tilt from 13.2 ± 4.9 degrees preoperatively to 0.8 ± 4.8 degrees at screw removal (p<0.001) [28].

The different bone pathologies influenced the correction rate. Chang et al. described a cohort with a mixed-etiology series of 37 patients and 63 ankles and demonstrated that underlying diagnosis was an independent predictor of correction velocity: CP ankles corrected at 0.90 ± 0.17 degrees/month, compared with 0.32 ± 0.06 degrees/month for HME, 0.24 ± 0.06 degrees/month for spina bifida, and 0.38 ± 0.11 degrees/month for the idiopathic subgroup (p=0.02) [19]. Dussa et al. (155 limbs, 104 children, five diagnostic groups) confirmed overall correction of the lateral distal tibial angle (LDTA) of 6.6 ± 1.0 degrees over 19.3 ± 9.6 months at an average velocity of 0.45 ± 0.08 degrees/month but found no significant differences in velocity among the five groups (p=0.293) [21].

Westberry et al. reported outcomes in 119 patients (189 extremities) with diverse pathological diagnoses (clubfoot 38%, CP/familial spastic paraparesis 15%, HME 8%, and syndromes/skeletal dysplasia 11%), demonstrating a mean LDTA improvement from 81.3 degrees preoperatively to 91.1 degrees at screw removal [31]. For OI, Franzone et al. reported a mean LDTA correction of 12.7 ± 8.5 degrees at a rate of 0.7 degrees/month in 12 distal tibial screw procedures, with one screw backout occurring in a figure-of-eight plate procedure, confirming that PETS can function effectively even in dysplastic OI bone with concurrent intramedullary rodding [14]. Similarly, Yilmaz et al. documented significant LDTA correction (73.9 ± 8.7 degrees to 86.1 ± 6.8 degrees; p<0.001) in a 6-patient/12-ankle subgroup with skeletal dysplasia [32].

Rebound after screw removal was the most clinically significant secondary finding in the ankle subgroup. Rupprecht et al. observed rebound valgus exceeding 5 degrees in 43% (6/14) of at-risk ankles in the HME population [28]. Re-epiphysiodesis was successfully performed in four patients [28]. In contrast, Westberry et al. reported rebound in only 5 of 189 extremities (3%) in their mixed series, and Tageldeen et al. reported a 2.5% rebound rate in Blount disease [15,31]. Recurrence was also high in the Chang et al. series, with 18 of 22 ankles (81.8%) demonstrating valgus recurrence after screw removal at a mean rate of 0.28 ± 0.08 degrees/month [19] (Table 4).

**Table 4:** Complications, Recurrence, and Secondary Procedures Incidence and nature of postoperative complications, including hardware-related events, rebound deformity rates following screw removal, and secondary surgical procedures performed across all anatomic subgroups and pathological diagnoses. Gp, group; LLD, limb-length discrepancy; MMC, myelomeningocele; MPTA, medial proximal tibial angle; NR, not reported; PETS, percutaneous epiphysiodesis using transphyseal screws; pt, patients; scr, screw.

Study	Complications (n, %)	Rebound (n,%)	Secondary procedures
Ankle			
Aurégan et al., 2011 [17]	None; no overcorrection	None	None (removal not required)
Chang et al., 2015 [19]	1 screw unremovable; 2 pt (3 ankles) not removed (maturity)	Valgus recurrence 18/22 ankles (82%); 0.28 ± 0.08°/mo	—
Driscoll et al., 2014 [20]	8/35 (23%) surgical: migration/overgrowth 4, breakage 2, symptomatic 2	0.20 ± 0.21°/mo; no physeal bar	Revision (1 broken screw)
Dussa et al., 2024 [21]	Recorded but not analyzed	Recorded but not analyzed	Foot-deformity correction (planned, at explant)
Franzone et al., 2022 [14]	1 screw backout (a plate, not the malleolar screw)	Not concludable	Plate revised to longer screws
Rupprecht et al., 2015 [28]	None (infection/neurovascular/implant/physeal damage)	6/14 at-risk ankles (43%); 0.37 ± 0.15°/mo	Re-epiphysiodesis 4 pt
Stevens and Belle, 1997 [30]	Superficial infection 1; premature removal 1 (MMC bursa)	5 pt overcorrected into varus (missed follow-up)	None
Westberry et al., 2020 [31]	Complicated extraction 69/189 (37%); 6 not fully removed	Rebound valgus 5 (3%)	Repeat screw 4, flatfoot recon 4, synovectomy 1
Yilmaz et al., 2014 [32]	1 broken screw at removal (whole cohort); no physeal closure	NR (screw subgroup)	None
Knee Coronal Plane							
Braga et al., 2022 [18]	Overcorrection 1; 1 screw unremovable; no breakage/bone bars	2 knees (excellent → good; MPTA p=0.049)	Corrective tibial osteotomy 2
Khoury et al., 2007 [23]	None in angular group; 1 recurvatum (LLD group)	6/13 removed physes (2°-15°)	Screw reinserted 1 (fibrous dysplasia)
Murphy et al., 2020 [26]	None; no implant failure	None; 7 pt radiographic overcorrection (not clinical)	None
Tageldeen et al., 2026 [15]	Reoperation 6 (7.5%); 0 overcorrection/failure/infection/arrest	2 (2.5%) > 5° varus	Repeat PETS 2
Knee Sagittal Plane							
Krajewski et al., 2025 [24]	1 per arm (retrograde screw migration; antegrade screw broke at removal)	Not assessed	—
Long et al., 2020 [9]	None surgery-related; knee pain 4/10 pt (implant-related 1)	Not assessed	Screw removal ~4 pt (concurrent)
Nazareth et al., 2020 [27]	Knee pain 2-scr 2/28 (7%), 1-scr 0/11; no arrest/overcorrection	NR	Hardware removal 16/47; 2 (2-scr) for recurrent crouch
Seth et al., 2024 [29]	Not specifically reported	Physeal-angle reversal with mid-third screw position	—
Hip							
Hsieh et al., 2019 [8]	Physis grew off screw 21/48 (44%); no osteonecrosis/chondrolysis	NR	Screw replacement 15/48; varus ± pelvic osteotomy 8/48
Hsu et al., 2020 [22]	Growing-off Gp1 16/37 (43%), Gp2 4/24 (17%); no physeal closure	Not assessed	Screw revision (growing-off)
Lee et al., 2016 [25]	None (no infection/fracture/osteonecrosis/chondrolysis); no bone bar	Not assessed	4/13 hips remained displaced

Surgical complications were predominantly hardware-related. Driscoll et al. reported postoperative complications in 23% (8/35) of medial malleolar screw ankles, including screw migration/overgrowth (n=4), hardware breakage (n=2), and symptomatic hardware (n=2) [20]. Westberry et al. found difficulty extracting their implants that required specialized instrumentation in 37% of extremities, where six screws (9% of complicated cases) could not be fully removed [31]. They found duration of implantation to be the strongest predictor of difficult extraction, with an average of 1.8 years in complicated cases versus 1.4 years in uncomplicated cases (p<0.01) [31]. No series reported permanent physeal closure or deep infection attributable to the screw (Table 4).

Knee Coronal Plane

Four studies (113 patients, 180 knees) reported outcomes of lateral proximal tibial and/or medial/lateral distal femoral PETS for coronal-plane deformities. All four focused on Blount disease or adolescent tibia vara, and all used single fully threaded cannulated screws at the proximal tibia (Table 3).

Tageldeen et al. enrolled 61 patients (80 knees) with juvenile and adolescent Blount disease and reported a mechanical axis deviation (MAD) correction rate of 3.66 ± 1.51 mm/month, achieving a mean MAD improvement from 43.8 ± 15.1 mm preoperatively to 0.7 ± 5.2 mm at implant removal (p<0.001) [15]. The patients also demonstrated a corresponding medial proximal tibial angle (MPTA) improvement from 77.0 ± 4.5 degrees to 86.3 ± 2.4 degrees (p<0.001). Rebound deformity occurred in only 2 of 80 knees (2.5%), and no overcorrection, hardware failure, or deep infection was observed [15] (Table 4).

Murphy et al. reported results in 9 patients (14 limbs) with juvenile and adolescent tibia vara at an average BMI of 31.7 kg/m^2^, demonstrating MAD improvement from a mean 49 mm to 0 mm (p<0.001) and MPTA from 81 degrees to 92 degrees (p<0.001) at a mean of 23 months [26]. No complications or implant failures were encountered. Braga et al. reported 90% surgical success (excellent in 12 of 20 knees, good in 6 of 20, and poor in 2 of 20) in adolescent tibia vara with a mean follow-up of 65 months to skeletal maturity, with rebound in 2 of 14 knees after removal and no implant breakage [18]. Khoury et al. reported pooled coronal-plane rates of 0.75 ± 0.45 degrees/month at the distal femur and 0.37 ± 0.34 degrees/month at the proximal tibia across a mixed pathological and idiopathic cohort; the 4 Blount limbs in the tibial subgroup averaged 0.79 degrees/month, exceeding the group mean [23].

Knee Sagittal Plane (ADFH)

Four studies (113 patients, 223 knees) addressed ADFH using transphyseal screws for fixed knee flexion deformities, predominantly in patients with CP. Outcomes in this group were measured by knee extension range of motion and distal femoral physeal angle (Table 3).

Seth et al. enrolled 36 patients (68 knees) with CP and other neuromuscular conditions, documenting a mean increase in the lateral distal femoral physeal angle from 93.0 ± 4.0 degrees at surgery to 102.4 ± 5.7 degrees at 12 months (delta 9.4 degrees, p<0.001) [29]. Nazareth et al. compared one-screw, two-screw, and plate-and-screw ADFH constructs in 47 patients with CP, MMC, and related conditions and found equivalent knee flexion correction across the groups (delta 12 degrees for both screw groups, p<0.006), with significantly lower postoperative knee pain in the two-screw group (7%) compared with the plate-and-screw group (63%, p=0.002) [27].

Long et al. (2020) reported radiographic improvement of 20.9 degrees in the posterior distal femoral angle at a mean 26-month follow-up in a 10-patient CP series, alongside significant improvements in clinical knee flexion contracture from -21.7 degrees to -3.8 degrees (p<0.001) [9]. Krajewski et al. found comparable clinical outcomes between antegrade and retrograde ADFH approaches (passive knee extension improvement of 6.4 degrees and 8.6 degrees, respectively; p>0.05 between groups), with the retrograde approach offering simplified hardware insertion and removal [24].

Coxa Valga

Three studies (65 patients, 122 hips) reported results of proximal femoral transphyseal screw-guided growth for coxa valga in CP. Outcome measures were the head-shaft angle (HSA), Reimers migration percentage (MP), acetabular index, and femoral anteversion angle (Table 3).

Lee et al. described an institutional series consisting of 9 children (13 hips) with spastic CP at GMFCS levels IV-V, demonstrating a mean HSA reduction of 11.9 degrees and MP improvement from 52.2% to 37.1% over 2 years (p<0.001 for 3-month to 1-year change) [25]. Hsieh et al. enrolled 24 patients (48 hips) and reported a mean HSA reduction of 13 ± 7 degrees and an MP reduction of 10 ± 11% at a mean 50-month follow-up [8].

Regression analysis identified longer follow-up duration and smaller preoperative MP as predictors of greater HSA correction [8]. Hips with baseline MP greater than or equal to 50% were substantially less likely to be stabilized by guided growth alone, with 8 of 16 (50%) ultimately requiring reconstructive surgery [8]. Hsu et al. demonstrated that screw placement within the medial physis predicts correction magnitude: Group 1 (medial quarter) achieved HSA reduction of 13.9 degrees vs. 8.7 degrees in Group 2 (middle quarter; p=0.003) but at the cost of a significantly higher rate of physeal growth-off (43.2% vs. 16.7%; p=0.038) [22].

The most consistent complication across the three hip series was progressive loss of epiphyseal screw purchase as continued physeal growth caused the screw tip to migrate out of the epiphysis. In Hsieh et al., this occurred in 21 of 48 hips (44%) at a mean of 28 months, necessitating screw replacement in 15 hips (31%) [8]. In Hsu et al., the rate ranged from 17% in the more centered Group 2 to 43% in the more medial Group 1 [22]. Younger patient age and more eccentric screw positioning were independent risk factors [22]. Importantly, no permanent physeal bar, chondrolysis, or avascular necrosis was documented in any of the three-hip series (Table 4).

Functional Outcomes

Formal functional outcome measures were reported in a minority of studies. Tageldeen et al. administered the Böstman knee score postoperatively, achieving a mean of 29.1 ± 0.9 (maximum 30), indicating excellent functional recovery in Blount disease [15]. Stevens and Belle reported patient/parent satisfaction in 22 of 31 ankle patients (71%), with 8 reporting no change and 1 expressing disappointment. Long et al. assessed the Pediatric Outcomes Data Collection Instrument (PODCI), Functional Mobility Scale (FMS), and Functional Assessment Questionnaire (FAQ) but found no statistically significant improvements in these global scores despite significant kinematic and clinical gains [9]. No other included study employed a validated patient-reported or functional outcome instrument.

Methodological Quality

Methodological quality was moderate and consistent with the retrospective evidence base. Among the 15 non-comparative case series, MINORS scores ranged from 8 to 12 of 16 (median 10). The 5 comparative studies scored 16 to 19 of 24 (median 17). Scores were limited primarily by the retrospective, single-center design (only one study collected data prospectively) and by the absence of any prospective sample-size calculation, blinded outcome adjudication in several series, and incomplete loss-to-follow-up reporting. Comparative studies were further limited by baseline non-equivalence between groups, most commonly in age, deformity severity, or follow-up duration (Tables 5, 6).

**Table 5:** Methodological Quality of Non-comparative Studies (MINORS) Item-level scoring for the 15 non-comparative studies was assessed using the methodological index for non-randomized studies (MINORS) criteria. Each of the 8 items was scored 0 (not reported), 1 (reported but inadequate), or 2 (reported and adequate), yielding a maximum possible score of 16. Items assessed include a clearly stated aim, inclusion of consecutive patients, prospective collection of data, endpoints appropriate to the aim, unbiased assessment of the endpoint, a follow-up period appropriate to the aim, loss to follow-up of less than 5%, and prospective calculation of study size. Maximum score for non-comparative studies = 16

Study	1	2	3	4	5	6	7	8	Total (out of 16)
Dussa et al., 2024 [21]	2	1	0	2	1	2	0	0	8
Franzone et al., 2022 [14]	2	1	0	2	1	2	1	0	9
Rupprecht et al., 2015 [28]	2	2	0	2	1	2	1	0	10
Stevens and Belle, 1997 [30]	2	2	0	2	2	2	1	0	11
Westberry et al., 2020 [31]	2	1	0	2	2	2	1	0	10
Yilmaz et al., 2014 [32]	2	1	0	2	1	1	1	0	8
Braga et al., 2022 [18]	2	2	0	2	1	2	2	0	11
Khoury et al., 2007 [23]	2	2	0	2	2	2	1	0	11
Murphy et al., 2020 [26]	2	2	0	2	1	1	1	0	9
Tageldeen et al., 2026 [15]	2	2	2	2	2	1	1	0	12
Seth et al., 2024 [29]	2	2	0	2	2	1	1	0	10
Hsieh et al., 2019 [8]	2	2	0	2	2	2	2	0	12
Lee et al., 2016 [25]	2	2	0	2	1	2	1	0	10

**Table 6:** Methodological Quality of Comparative Studies (MINORS) Item-level scoring for the five comparative studies was assessed using the methodological index for non-randomized studies (MINORS) criteria. Scoring includes the eight non-comparative items plus four additional items: adequate control group, contemporary groups, baseline equivalence of groups, and adequate statistical analyses. Each item was scored 0 (not reported), 1 (reported but inadequate), or 2 (reported and adequate), yielding a maximum possible score of 24. Item-level scoring for the five comparative studies was assessed using the methodological index for non-randomized studies (MINORS) criteria. Scoring includes the eight non-comparative items plus four additional items: adequate control group, contemporary groups, baseline equivalence of groups, and adequate statistical analyses. Each item was scored 0 (not reported), 1 (reported but inadequate), or 2 (reported and adequate), yielding a maximum possible score of 24.

Study	1	2	3	4	5	6	7	8	9	10	11	12	Total (out of 24)
Driscoll et al., 2014 [20]	2	2	0	2	1	2	1	0	2	2	1	2	17
Long et al., 2020 [9]	2	2	0	2	1	1	1	0	2	2	1	2	16
Nazareth et al., 2020 [27]	2	2	0	2	1	1	1	0	2	2	1	2	16
Krajewski et al., 2025 [24]	2	2	0	2	1	2	1	0	2	2	1	2	17
Hsu et al., 2020 [22]	2	2	0	2	2	2	2	0	1	2	2	2	19

Discussion

This systematic review is the first to comprehensively synthesize the outcomes of transphyseal screw hemiepiphysiodesis across a spectrum of pathological bone conditions and anatomic sites in skeletally immature patients. Overall, we found PETS to be broadly effective in this population. Statistically and clinically significant angular correction is achievable across Blount disease, HME, CP, MMC, skeletal dysplasia, OI, and mixed pathological diagnoses, at every anatomic location studied. Importantly, no included study documented permanent physeal closure attributable to the transphyseal screw, and no case of neurovascular injury was reported.

These findings are based upon the existing PETS literature. Prior systematic reviews demonstrated that PETS achieves higher angular correction rates than tension-band plating for coronal-plane knee deformities in predominantly idiopathic populations. The present data confirm that this efficiency advantage is preserved in pathological cohorts, but they also reveal that pathological biology introduces variations in correction velocity and timing that have important surgical planning implications.

Correction rates vary significantly by underlying diagnosis, a finding with direct implications for predicting time to correction and planning hardware removal. Chang et al. provided the clearest demonstration, reporting an approximately 3.5-fold range in tibiotalar angle correction rate from 0.24 degrees/month in spina bifida to 0.90 degrees/month in CP, with HME and mixed categories falling between those extremes [24]. Dussa et al. confirmed etiology-dependent heterogeneity in absolute correction values, though their larger analysis found no statistically significant difference in mean velocity among the five groups (p=0.293), likely reflecting the confounding effects of age, initial deformity severity, and treatment duration.

The biological mechanisms underlying these differences are not fully established but likely include alterations in physeal cell density and proliferative capacity [33]. In CP, spasticity-associated compressive loading and relative physeal softening may enhance the Hueter-Volkmann growth-inhibition effect, accelerating correction [34,35]. In spina bifida, flaccid paralysis and resulting mechanical unloading may reduce the compressive stimulus needed for efficient guided growth [36]. In bone dysplasias, the intrinsic disorganization of the growth plate growth architecture may slow the growth response even when physeal compression is adequate [37].

These distinctions carry practical importance: a surgeon operating on a 12-year-old with HME ankle valgus should anticipate a substantially different time to correction than one treating a child with CP ankle valgus of equivalent severity, and the removal threshold and follow-up interval should be adjusted accordingly.

The incidence of rebound deformity after screw removal was highly variable, ranging from 2.5% in Blount disease to 43% in HME ankle valgus and 81.8% in the Chang series [15,20,24]. These findings reflect differences in etiology, physeal maturity at removal, and the duration of the post-removal follow-up window. Several studies have suggested that rebound deformity occurs when screws are removed before physeal closure. In the Rupprecht series, HME patients with closed physes at the time of removal had no recurrence, compared with a 43% rebound rate in those with open physes [20].

Despite these findings, rebound can be managed successfully. For instance, repeated epiphysiodesis was successfully performed in 4 of 4 HME patients with rebound and adequate residual growth in the Rupprecht series, and Tageldeen et al. retreated 2 of 2 rebound Blount knees with repeat PETS [15,20,38].

These observations suggest that the strategy of deliberate slight overcorrection before screw removal may not be necessary in all pathological conditions. Tageldeen et al. explicitly targeted neutral alignment at removal and achieved a very low rebound rate, consistent with emerging data suggesting that direct physeal compression by PETS may produce more definitive physeal modulation than the tension-band mechanism [15]. Further prospective studies assessing the optimal removal threshold in each pathological diagnosis are warranted.

The specific hardware complication profile of transphyseal screws differs importantly from that of tension-band plates. The most prevalent complication in the ankle cohort was difficult screw extraction, occurring in 37% of cases in the Westberry series [22]. Contributing factors included younger patient age at implantation (mean 11.2 years in complicated cases vs. 11.9 years in uncomplicated cases) and longer implantation duration (1.8 vs. 1.4 years; p<0.01) [22]. The screw diameter also mattered, as the 3.5 mm screws were more difficult to remove than 4.0 mm screws [22].

Driscoll et al. found hardware-related surgical complications in 23% of screw ankles versus 4% of tension-band plate ankles, suggesting that the PETS technique carries a hardware burden that should be clearly communicated to families preoperatively [20]. Despite this, the rate of symptomatic hardware requiring reoperation was low (5.7% in the Driscoll screw group), and no cases of intra-articular migration or neurovascular injury were documented [20].

When assessing proximal femoral implantation, the characteristic complication was progressive loss of epiphyseal screw purchase as continued physeal growth caused the screw tip to migrate out of the epiphysis, occurring in 17%-44% of hips across the three CP series [8,22,25]. These findings suggest a different mechanism for hardware failure, in which the relative growth velocity of the proximal femoral physis outpaces the stable implant, creating progressive shortening of the intraepiphyseal thread purchase [31,39]. Associated risk factors included younger age, more eccentric screw position, and a larger preoperative HSA [31]. The authors suggested that positioning the screw across the middle quarter rather than the most medial portion of the physis in younger children reduces the growing-off rate (from 43% to 17%), with a modest reduction in the varus correction magnitude [31].

Limitations

This review has several important limitations. The evidence base consists entirely of level II-IV non-randomized studies, and the majority are retrospective single-center case series with inherent selection, measurement, and reporting biases. Outcome heterogeneity precluded pooled quantitative synthesis and limits the precision of etiology-specific conclusions.

Formal MINORS scores were limited to moderately low quality in most studies, reflecting predominantly retrospective design and the frequent absence of validated functional outcomes, prospective data collection, and loss-to-follow-up reporting. Finally, a formal assessment of publication bias was not possible given the descriptive nature of the review.

## Conclusions

Transphyseal screw hemiepiphysiodesis is effective and reversible across a broad spectrum of pathological bone conditions. Correction rates are diagnosis-dependent, ranging from 0.24 degrees/month in spina bifida to 0.90 degrees/month in CP at the ankle, with rebound most pronounced in HME after premature removal. In Blount disease, MAD correction averaged 3.66 mm/month with low rebound when neutral alignment was targeted at removal. For ADFH, anterior physeal screw placement is critical for sustained sagittal correction.

In CP coxa valga, guided growth provides effective hip containment in moderately displaced hips but carries a notable rate of physeal growth-off requiring revision surgery while preserving overall physeal viability. Permanent physeal arrest was not documented in any series. The evidence base remains limited to retrospective studies; prospective multicenter trials are needed to optimize implantation duration and removal timing across pathological etiologies.
